# Educational Limitations of ChatGPT in Neurosurgery Board Preparation

**DOI:** 10.7759/cureus.58639

**Published:** 2024-04-20

**Authors:** Andrew Y Powers, Martin G McCandless, Philipp Taussky, Rafael A Vega, Max S Shutran, Ziev B Moses

**Affiliations:** 1 Neurosurgery, Beth Israel Deaconess Medical Center, Harvard Medical School, Boston, USA; 2 Neurosurgery, University of Mississippi Medical Center, Jackson, USA

**Keywords:** neurosurgery, medical education, machine learning, large language model, artificial intelligence

## Abstract

Objective

This study evaluated the potential of Chat Generative Pre-trained Transformer (ChatGPT) as an educational tool for neurosurgery residents preparing for the American Board of Neurological Surgery (ABNS) primary examination.

Methods

Non-imaging questions from the Congress of Neurological Surgeons (CNS) Self-Assessment in Neurological Surgery (SANS) online question bank were input into ChatGPT. Accuracy was evaluated and compared to human performance across subcategories. To quantify ChatGPT’s educational potential, the concordance and insight of explanations were assessed by multiple neurosurgical faculty. Associations among these metrics as well as question length were evaluated.

Results

ChatGPT had an accuracy of 50.4% (1,068/2,120), with the highest and lowest accuracies in the pharmacology (81.2%, 13/16) and vascular (32.9%, 91/277) subcategories, respectively. ChatGPT performed worse than humans overall, as well as in the functional, other, peripheral, radiology, spine, trauma, tumor, and vascular subcategories. There were no subjects in which ChatGPT performed better than humans and its accuracy was below that required to pass the exam. The mean concordance was 93.4% (198/212) and the mean insight score was 2.7. Accuracy was negatively associated with question length (R^2^=0.29, p=0.03) but positively associated with both concordance (p<0.001, q<0.001) and insight (p<0.001, q<0.001).

Conclusions

The current study provides the largest and most comprehensive assessment of the accuracy and explanatory quality of ChatGPT in answering ABNS primary exam questions. The findings demonstrate shortcomings regarding ChatGPT’s ability to pass, let alone teach, the neurosurgical boards.

## Introduction

Evaluating neurosurgical standards of care necessitates rigorous examination to uphold the integrity and quality of patient treatment and safety [1]. The American Board of Neurological Surgery (ABNS) is a critical entity, which oversees the certification of neurosurgeons in the United States. One of the components of this certification process is the ABNS primary exam, a written exam that assesses the fundamental knowledge and skills necessary for a resident physician to proceed with neurosurgery training. It emphasizes clinical knowledge, judgment, and decision-making in the context of neuroanatomy, neuropathology, clinical neurology, neuroradiology, neurocritical care, and neurosurgical techniques [2]. This annual exam functions as a milestone in the pathway to becoming board-certified and has evolved over time to enhance its objectivity, validity, and relevance to contemporary neurosurgical practice [1,3].

In the past year, advances in artificial intelligence, specifically natural language processing, have led to the emergence of state-of-the-art large language models, which are able to perform a variety of text-based tasks, including solving math problems, taking standardized tests, coding, and even writing poetry [4-7]. The release of perhaps the most well-known of these models, the Chat Generative Pre-trained Transformer (ChatGPT) (OpenAI, 2022), sent the medical community into a frenetic search for ways in which to incorporate this innovative technology [8].

There has been great interest in benchmarking the level of medical “understanding” of large language models, resulting in the publication of numerous studies evaluating ChatGPT’s performance on the United States Medical Licensing Examination and many medical subspecialty exams [9-16].

Additionally, ChatGPT has shown potential in explaining medical reasoning and analyzing clinical cases, introducing unprecedented possibilities for medical education [10,17]. The use of ChatGPT in the context of neurosurgical education, however, is still a nascent field. A recent systematic review of ChatGPT’s potential as an educational tool raised both ethical and practical concerns [18]. While a couple of initial studies have investigated the performance of ChatGPT on practice neurosurgery board questions, these studies use only a fraction of available questions and do not explore ChatGPT’s ability to explain its reasoning, a critical component of evaluating both its potential use as an educational tool [19,20].

The current study provides the largest and most comprehensive assessment of the accuracy and explanatory quality of ChatGPT in answering ABNS primary exam questions. The findings demonstrate shortcomings regarding ChatGPT’s ability to pass, let alone teach, the neurosurgical boards.

## Materials and methods

Data collection

The Congress of Neurological Surgeons (CNS) Self-Assessment in Neurological Surgery (SANS) online question bank, parts one through four, was used for this study. This question bank is comprised of questions that were previously used in the ABNS primary exam and are divided into subjects, including American College of Graduate Medical Education (ACGME), anatomy, functional, fundamentals, neurobiology, other, pain, pathology, pediatrics, peripheral, pharmacology, radiology, spine, statistics, trauma, tumor, and vascular. For each subject, aggregate human accuracy statistics were obtained from the SANS website; however, individual data were not publicly available.

Questions that included imaging as well as those with a number of answer choices other than five were removed. The remaining questions were input into ChatGPT with the preceding prompt “Select the single best answer to the following multiple-choice question.” The response was recorded and compared with the correct answer for accuracy.

ChatGPT was then prompted to give an explanation with “Why did you choose that answer?” A 10% (212/2,120) subject-stratified random sample of explanations was evaluated for concordance and insight as described previously in the literature [10]. Both metrics serve as proxies for language model “understanding” and may also serve as indicators of educational potential. A concordant explanation is defined as one that is not self-contradictory, a highly desirable educational quality, as self-contradictory explanations cause confusion and necessarily include false information. Insight is defined as a factually true statement that does not simply define a term in the question, requires deduction or information not listed in the question, and is distinct from other insights in the explanation. The quantity of insights may serve as a measure of the number of potential learning opportunities provided by the explanation. For each sampled explanation, two neurosurgical faculty independently evaluated whether it was concordant or not and counted the number of insights. A senior, board-certified neurosurgeon arbitrated any score mismatches.

This study also explored the role of prompt length on ChatGPT’s accuracy. Language models interpret prompts as sequences of standardized “tokens,” which are words, word fragments, or individual symbols [21]. To calculate question length, each question was input into a tokenizer, which separates prompts into tokens, similar to how ChatGPT parses prompts [22].

This study did not require institutional review board approval, as there were no human or animal study participants. Additionally, this research did not receive any specific grant from funding agencies in the public, commercial, or not-for-profit sectors.

Statistical analysis

ChatGPT and human performance were compared using chi-squared tests. Question length and accuracy were compared for each subject, with the overall association evaluated by subject-stratified linear regression. The inter-rater reliability of concordance and insight scores was evaluated using Cohen’s kappa coefficient. Concordance and insight were compared between correctly and incorrectly answered questions using Mann-Whitney U tests. Correction for multiple hypothesis testing was performed for each set of tests using the Benjamini-Hochberg procedure with a predetermined false discovery rate of 0.05. Adjusted p-values are denoted as q-values. All analyses were implemented using R (v4.2.1; R Core Team, 2022).

## Results

Of an initial 2,816 questions, 690 (24.5%) involved imaging and were discarded. A further six (0.2%) questions had a number of answer choices other than five and were removed, leaving a total of 2,120 (75.3%) questions for input into ChatGPT. The plurality of questions answered by ChatGPT (13.1%, 277/2,120) and humans (12.6%, 355/2,816) were both in the vascular subcategory.

**Figure 1:**
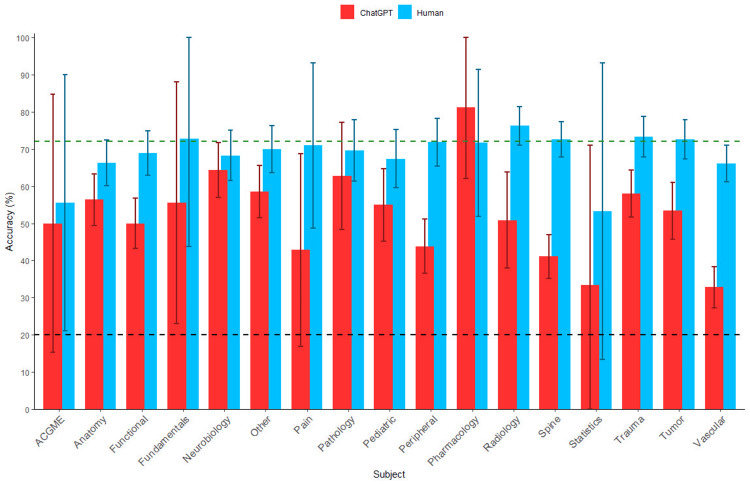
Comparison of ChatGPT and Human Accuracy, Stratified by Subject ChatGPT (red) and mean human (blue) percent accuracy are compared across subjects, with the error bars representing 95% confidence intervals. The dotted green line represents the minimum passing percentage in 2023 (72%). The dotted black line represents the expected percent performance obtained through random selection (20%). ACGME, American College of Graduate Medical Education; ChatGPT, Chat Generative Pre-trained Transformer

ChatGPT’s overall accuracy was 50.4% (1,068/2,120) with the highest and lowest accuracies in the pharmacology (81.2%, 13/16) and vascular (32.9%, 91/277) subcategories, respectively. In comparison, overall mean human accuracy was 70.3% (counts unavailable) with the highest and lowest accuracies in the radiology (76.2%, counts unavailable) and statistics (53.2%, counts unavailable) subcategories, respectively. Compared to humans, ChatGPT performed significantly worse overall (p<0.001, q<0.001) as well as in the functional, other, peripheral, radiology, spine, trauma, tumor, and vascular subcategories. There were no subjects in which ChatGPT performed significantly better than humans (Figure 1; Table 1).

**Table 1:** Comparison of ChatGPT and Human Accuracy, Stratified by Subject *Percentages may not sum to 100 due to rounding. †Significant without correction for multiple hypothesis testing (p<0.05). ‡Significant following correction for multiple hypothesis testing (q<0.05). Subject distributions are represented by question counts with corresponding percentages. Mean accuracy is represented by percentages with 95% confidence intervals. Significant differences are represented by p- and respective corrected q-values. ACGME: American College of Graduate Medical Education, ChatGPT: Chat Generative Pre-trained Transformer

	Subject Distribution^*^		Accuracy (%)			
Subject	ChatGPT	Human	ChatGPT	Human	p-value	q-value
Overall	2120 (100.0)	2816 (100.0)	50.4 (48.2, 52.5)	70.3 (68.7, 72.0)	<0.001^†^	<0.001^‡^
ACGME	8 (0.4)	8 (0.3)	50.0 (15.4, 84.6)	55.6 (21.2, 90.0)	>0.99	>0.99
Anatomy	197 (9.3)	228 (8.1)	56.3 (49.4, 63.3)	66.3 (60.1, 72.4)	0.05^†^	0.08
Functional	208 (9.8)	232 (8.2)	50.0 (43.2, 56.8)	68.9 (62.9, 74.8)	<0.001^†^	<0.001^‡^
Fundamentals	9 (0.4)	9 (0.3)	55.6 (23.1, 88.0)	72.8 (43.7, 100.0)	0.79	0.89
Neurobiology	160 (7.5)	183 (6.5)	64.4 (57.0, 71.8)	68.3 (61.5, 75.0)	0.52	0.69
Other	188 (8.9)	200 (7.1)	58.5 (51.5, 65.6)	70.0 (63.6, 76.3)	0.02^†^	0.05^‡^
Pain	14 (0.7)	16 (0.6)	42.9 (16.9, 68.8)	71.0 (48.7, 93.2)	0.24	0.35
Pathology	43 (2.0)	119 (4.2)	62.8 (48.3, 77.2)	69.6 (61.3, 77.8)	0.53	0.69
Pediatrics	100 (4.7)	138 (4.9)	55.0 (45.2, 64.8)	67.4 (59.5, 75.2)	0.07	0.12
Peripheral	178 (8.4)	187 (6.6)	43.8 (36.5, 51.1)	71.8 (65.4, 78.3)	<0.001^†^	<0.001^‡^
Pharmacology	16 (0.8)	20 (0.7)	81.2 (62.1, 100.0)	71.7 (51.9, 91.4)	0.78	0.89
Radiology	57 (2.7)	259 (9.2)	50.9 (37.9, 63.9)	76.2 (71.1, 81.4)	<0.001^†^	<0.001^‡^
Spine	263 (12.4)	333 (11.8)	41.1 (35.1, 47.0)	72.6 (67.8, 77.3)	<0.001^†^	<0.001^‡^
Statistics	6 (0.3)	6 (0.2)	33.3 (0.0, 71.1)	53.2 (13.3, 93.2)	0.91	0.96
Trauma	233 (11.0)	252 (8.9)	57.9 (51.6, 64.3)	73.4 (67.9, 78.8)	<0.001^†^	<0.001^‡^
Tumor	163 (7.7)	271 (9.6)	53.4 (45.7, 61.0)	72.5 (67.2, 77.9)	<0.001^†^	<0.001^‡^
Vascular	277 (13.1)	355 (12.6)	32.9 (27.3, 38.4)	66.1 (61.2, 71.0)	<0.001^†^	<0.001^‡^

**Figure 2:**
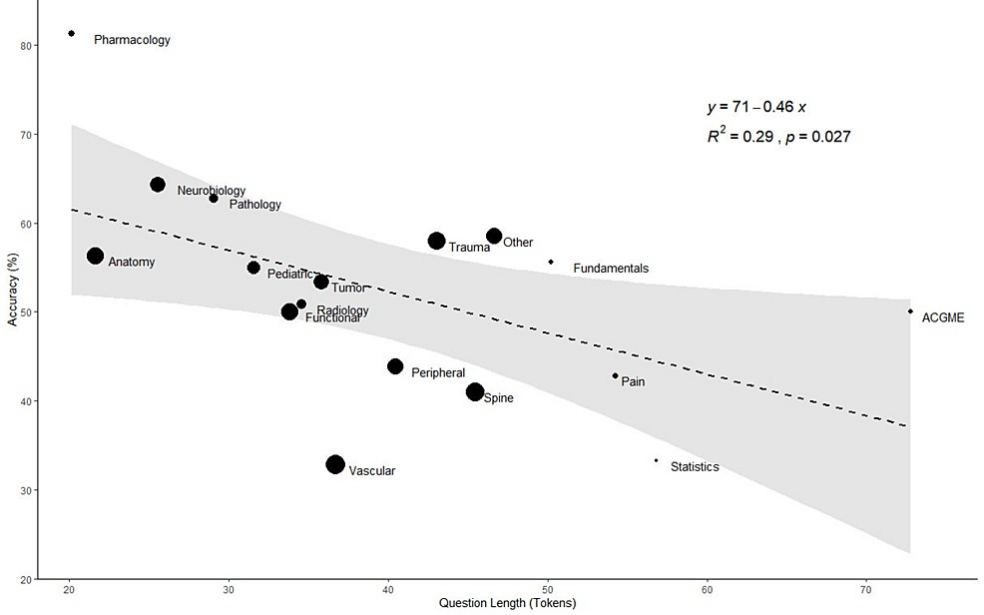
ChatGPT’s Accuracy Compared to Question Length, Stratified by Subject ChatGPT’s percent accuracy is compared to token length and stratified across subjects, which are denoted by points that are proportional in size to the number of questions ChatGPT answered within those subjects. The black dotted line represents the subject-stratified, linearly approximated relationship between question length and ChatGPT’s percent accuracy, while the gray-shaded region represents the 95% confidence interval of this linearly approximated relationship. ChatGPT: Chat Generative Pre-trained Transformer

On average, the shortest and longest questions belonged to the pharmacology (20.1 tokens) and ACGME (72.8 tokens) subcategories, respectively. ChatGPT’s accuracy demonstrated a significant negative association with question length when generating a subject-stratified linear regression over these values (R^2^=0.29, p=0.03) (Figure 2).

A total of 212 explanations were randomly selected with subject-proportionate representation for independent evaluation by two neurosurgical attendings. Only eight (3.8%) responses required arbitration by a third neurosurgical attending due to differences in assigned concordance (κ=0.93) or insight (κ=0.96) scores.

The rate of concordance was 93.4% (198/212), while the mean insight score was 2.7. Correctly answering a question demonstrated a significant positive association with both concordance (p<0.001, q<0.001) and insight (p<0.001, q<0.001) (Table 2).

**Table 2:** Explanatory Quality, Stratified by Accuracy *Significant without correction for multiple hypothesis testing (p<0.05). †Significant following correction for multiple hypothesis testing (q<0.05). Mean concordance percentages and mean insight scores, each with 95% confidence intervals, are shown overall as well as for correctly and incorrectly answered questions. Significant differences are shown with p- and respective corrected q-values.

	Overall (n=212, 100%)	Correct (n=111, 52.4%)	Incorrect (n=101, 47.6%)	p-value	q-value
Concordance (%)	93.4 (90.1, 96.7)	99.1 (97.3, 100.0)	87.1 (80.6, 93.7)	<0.001^*^	<0.001^†^
Insight	2.7 (2.5, 2.8)	2.9 (2.8, 3.1)	2.4 (2.2, 2.5)	<0.001^*^	<0.001^†^

## Discussion

Accuracy

When assessed on more than 2,000 questions using all parts of the CNS SANS question bank, ChatGPT achieved a fairly unimpressive overall accuracy of 50.4% (1,068/2,120). Our findings corroborate those of Hopkins et al., who found a similar accuracy of 54.9% (262/477) using non-imaging questions from another question bank [19]. In contrast, Ali et al. reported a much higher accuracy of 73.4% (367/500) using both imaging and non-imaging questions from part one of the CNS SANS question bank [20]. Notably, part one of the question bank has disproportionately few vascular questions, which we found was the subject in which ChatGPT had the worst performance; however, other factors such as the version of ChatGPT used and prompt engineering likely also play a role in the difference in reported performance. High sensitivity to the specific wording of input questions reveals a key limitation in ChatGPT’s educational utility, as learners may not always pose questions in a way that triggers an optimal response.

The accuracy required to pass the neurosurgery board exam in 2023 was 72%. The overall mean human accuracy of 70.3% (counts unavailable) across all parts of the question bank was similar to this cutoff and significantly higher than that of ChatGPT. This average human performance includes those of more junior residents who may be several years from taking the board exam for credit. Additionally, residents use the question bank as a study tool, meaning that the expected level of performance at the end of using the question bank and at the time of the actual exam would be higher than the average performance measured when using the question bank. In contrast, we would not expect ChatGPT to perform differently on the real board exam. Beyond this performance gap, the version of ChatGPT used in this study cannot interpret images, which are present in nearly a quarter of the prompts in the CNS SANS question bank and represent a critical component of both the real exam and neurosurgical practice.

In addition to performing worse overall, ChatGPT also performed significantly worse than humans in the functional, other, peripheral, radiology, spine, trauma, tumor, and vascular categories. ChatGPT did not perform significantly differently than humans in the ACGME, fundamentals, pain, pharmacology, and statistics categories, as each of these subjects had fewer than 20 questions, providing minimal statistical power to detect such differences. ChatGPT also did not perform significantly worse in the anatomy, neurobiology, pathology, or pediatrics categories, possibly because information regarding these subjects was better represented in its training data.

Corroborating previous reports, subjects with longer average question lengths were generally those on which ChatGPT performed worse [20]. As with humans, ChatGPT’s differential performance across subjects may reveal differences in the ability to handle subject-specific complexity. Differences in question length across subjects may also in part reflect stylistic differences among question writers.

Explanatory quality

A recent report by Mannam et al. introduced a novel scoring system for explanatory quality when answering neurosurgery board questions; however, the significance and reproducibility of these scores remain unclear given their recent publication [23]. Concordance and insight are metrics, which have been used previously to assess the explanatory quality of large language models [10]. The high inter-rater reliability of these scores demonstrates their reproducibility in the current study. The positive association found between correctly answering a question and both concordance and insight indicate that ChatGPT has a better understanding of correctly answered questions. The number of insights provides an important benchmark against which to compare the educational value of future language models.

Although concordance is generally a desirable quality, even incorrectly answered questions were associated with high concordance (87.1%, 88/101) in this study. The lack of self-contradiction in a concordant explanation may seem “more confident,” illustrating ChatGPT’s potential to mislead trainees who do not know the correct answer with a superficially confident, but ultimately incorrect, explanation.

Strengths and limitations

The current study faces several limitations. The exclusion of imaging questions reduces the generalizability of the results due to the difference in complexity from the real board exam. The smaller sample of explanations evaluated for concordance and insight scores also precludes analysis of subject-specific educational quality. Finally, different results may be obtained by using different ChatGPT versions, alternative prompt engineering, or other large language models such as GPT-4 (OpenAI, 2023) or Bard (Google, 2023) [5,24].

Despite these limitations, this study contributes significantly to existing literature on this topic. The inclusion of all parts of the CNS SANS question bank more than quadruples the sample sizes of previous studies, yielding more precise performance metrics across a wider variety of questions as well as allowing for more granular, subject-specific analyses. Using token, rather than word, counts more accurately reflects ChatGPT’s representation of question length. Most notably, the evaluation of explanatory quality through concordance and insight provides a deeper understanding of ChatGPT’s capabilities and shortcomings as an educational tool beyond assessments of accuracy alone. This process was conducted by corroborating scores of multiple neurosurgical faculty to ensure consistency and mitigate bias. Finally, the use of non-parametric statistical tests and correction for multiple hypothesis testing minimizes the likelihood of spurious positive findings [25].

## Conclusions

While future improvements will undoubtedly, and perhaps sooner than we expect, allow successors of ChatGPT to assist in neurosurgical education, present models suffer from substantial deficiencies. The current study provides the most extensive analysis to date of ChatGPT’s accuracy on ABNS primary exam questions as well as provides an exploration of its explanatory quality, a key facet in the evaluation of its ability to educate neurosurgical trainees.

Future research will likely involve multimodal models that can synthesize multiple types of input data, such as imaging and text, to simulate more closely the clinical reasoning performed by neurosurgeons. Extending our evaluation of explanatory quality to a larger sample of questions can reveal the neurosurgical subcategories in which ChatGPT would most benefit from interacting with additional training material. Although much excitement surrounds recent advancements in artificial intelligence, particularly with respect to large language models, studies such as ours underscore the current gaps in both accuracy and explanatory quality within highly specialized domains such as neurosurgery that limit their educational utility.
